# Effects of sweet cassava polysaccharide extracts on endurance exercise in rats

**DOI:** 10.1186/1550-2783-10-18

**Published:** 2013-03-28

**Authors:** Chia Hung Yen, Te Hung Tsao, Cheng Uan Huang, Chang Bin Yang, Chung Sheng Kuo

**Affiliations:** 1 Department of Biological Science and Technology, National Pingtung University of Science & Technology, Pingtung, Taiwan; 2 Physical Education Section of General Education, National Sun Yat-Sen University, No. 70, Lienhai Rd., Kaohsiung City, 80424, Taiwan; 3 Department of Recreation Sport and Health Promotion, National Pingtung University of Science & Technology, Pingtung, Taiwan; 4 Department of Physical Education, National Dong Hwa University, HuaLien, Taiwan; 5 Chang Gung University of Science and Technology, Taoyuan, Taiwan

**Keywords:** Exhaustive running, Muscle glycogen, Blood glucose, Free fatty acids

## Abstract

**Background:**

Sweet cassava tubers have abundant carbohydrates consisting of monosaccharides and polysaccharides. In addition, polysaccharides extracted from plants improve sports performance, according to recent studies. We therefore examined whether the administration of sweet cassava polysaccharides (SCPs) benefited endurance performance in rats

**Methods:**

Male Sprague–Dawley rats (n = 30, 7 weeks old) were divided into three groups: control (C), exercise (Ex), and exercise plus SCPs administration (ExSCP) (at a dose of 500 mg/kg body weight by gastric intubation for six days in addition to standard rat food and water). An exercise program was implemented in the Ex and ExSCP groups for five days (with no exercise on the sixth day), and then all rats were sacrificed to determine the glycogen content of the gastrocnemius and soleus muscles, and the blood metabolites after the ExSCP and Ex groups had completed exhaustive running.

**Results:**

The running time to exhaustion of the ExSCP group was significantly longer than that of the Ex group by 49% (64 vs. 43 min). After running to exhaustion, it was seen that although the glycogen content in the soleus and gastrocnemius muscles of the Ex and ExSCP groups was lower compared to the C group, values in the ExSCP group were significantly higher than in the Ex group (*p* > 0.05). In addition, blood glucose and free fatty acid (FFA) levels were significantly higher in the ExSCP than in the Ex group (*p* > 0.05). However, no significant differences for blood glucose or FFA were found between the ExSCP and C groups.

**Conclusions:**

SCP supplementation can prolong exercise endurance in rats. Higher muscle glycogen levels and stable glucose and FFA concentrations in the circulation contributed to the prolonged time to exhaustion.

## Background

Sweet cassava is a major food or food ingredient in many countries. The composition of this tuber is 38% carbohydrate and 60% water [1]. A few studies [2-4] have indicated that the carbohydrates in cassava tubers contain monosaccharides (fructose, arabinose, and galactose) and polysaccharides. It has been reported that the intake of high-carbohydrate foods increases muscle glycogen content, which can prolong exercise time and delay fatigue [5,6]. Generally speaking, many sports, such as soccer, tennis, and track and field events, require athletes to compete repeatedly within the space of a few days. In addition, athletes train almost every day. If an athlete can maintain muscle glycogen via dietary supplementation, he/she can recover efficiently and engage in subsequent training or competition. Consequently, studies have examined the effects of regimens and substance supplementation on muscle glycogen and sports performance, for example, carbohydrate loading [7,8] and consumption of fenugreek seeds [9].

Recently, several studies have indicated that extracted polysaccharides provide the following benefits: enhancing muscle glycogen and sports performance, extending endurance times, resistance to fatigue, decreasing oxidative stress after strenuous exercise [10-12], and detoxifying the body [13]. Although sweet cassava is a staple food in many countries, and the literature indicates that it contains abundant carbohydrates and seems beneficial for sports performance, no study has reported the effects of sweet cassava or its extracted polysaccharides on sports performance. Therefore, the aim of this study was to examine the effects of sweet cassava polysaccharides (SCPs) on sports performance using a rat model. In addition to looking at exercise duration times, blood metabolites, such as free fatty acids (FFAs), blood glucose, and insulin, were measured. We hypothesized that SCP supplementation would increase muscle glycogen and prolong the running time to exhaustion.

## Materials

Male Sprague–Dawley (SD) rats (five weeks old and weighting 180~200 g) were maintained at a temperature of 24 ± 1°C in humidity-controlled conditions (45%~55%) with a 12-h light/dark schedule (lights on at 0600) and were allowed food and water *ad libitum*. Thirty SD rats were divided into three groups (10 rats/group): control (C), exercise (Ex), and exercise with SCP supplementation (ExSCP). The sample size in this study was decided by our pilot experiment. The dose and period of SCP supplementation were the same as the current study. Only the difference was that there were four rats in each Ex and ExSCP groups. An a priori statistical power analysis using the endurance performance as the primary result showed that eight rats in each group would provide adequate statistical power (0.8) to detect 10% difference in the running time to exhaustion (G*Power, Franz Faul, Kiel University, Germany). The supplementation experiment extended for a five-day period that began after an acclimatization period of one week. Test animals were twice placed on a rat treadmill (with at least a two-day interval to avoid a training effect) for 10 min at 10 m/min during acclimatization. The food was a standard rat chow (Fwusow, Taichung, Taiwan) mainly consisting mainly of carbohydrates (52%), protein (23.5%), fat (4.5%), water (12%), ash (10%), and fiber (8%). The average intake weights of the rat chow during the experimental period were 30.9 ± 2.2, 37.4 ± 3.7, and 36.6 ± 3.3 g/day/rat for the C, Ex, and ExSCP groups respectively, with the last two groups consuming significantly more than the first group. The use of a rat model in this study was approved and conducted under the guidelines of the Animal Studies Committee of National Pingtung University of Science and Technology.

### SCP preparation and dosage

**Figure 1:**
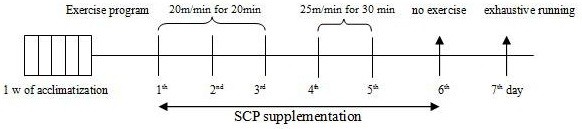
Overview of the experimental procedure.

The methods used in Charles and Huang [13] were adopted for isolating and preparing SCPs. The methods and procedures employed were, briefly, as follows: pellets were ground into cassava flour after preparatory procedures (i.e. the sweet cassava tuber was washed, peeled, and pelletized). The mixtures (250 g cassava flour with 500–750 g of water) were centrifuged at 14,300 g at 4°C for 20 min and the supernatants removed. Then, crude mucilage was produced when the supernatant was filtered, concentrated, and lyophilized. Crude polysaccharides were fractioned by anion exchange chromatography with elution by NaCl at different concentrations (0.5, 1.0, 2.0, and 3.0 ml). The SCP was purified by Sephacryl S-400/HR gel filtration chromatography after being pooled, concentrated, desalted, and freeze-dried. Test animals were fed a dose of 500 mg SCP/kg body weight/day. SCPs were given by gastric intubation in two 250 mg/kg doses; one after the morning exercise and the other in the evening at approximately 1700–1800. The dosage of SCP was determined by the rat’s daily weight measurement in the morning, and the SCP was mixed with physiological saline at 100 mg/ml. On the sixth day, the same supplementation times were used as had been on the previous five days, but there was no exercise. Exhaustive running was completed on the morning of the seventh day after overnight fast, and gastrocnemius and soleus muscles, as well as blood samples from all rats were collected after anesthetization and sacrifice (Figure 1).

### Exercise model

After one week of acclimatization, the Ex and ExSCP groups had one exercise bout each day for five days. The speed and duration of the first three days and the final two days were 20 m/min for 20 min and 25 m/min for 30 min respectively. Rats in the C group maintained their daily cage lifestyle without exercise training, but they were placed alongside the rat treadmill while the other two groups exercised. In this study, the speed in the exhaustive exercise model (30 m/min with 0% gradient) was selected using a study of Brooks and White [14], who used 30 m/min with a 10% gradient (70%~75% VO_2_max). The rats were motivated to run by gentle prodding with a nylon brush to the point of exhaustion, which was determined by an animal's loss of righting reflex when turned on its back.

### Glycogen and blood analysis

After the Ex and ExSCP groups had completed the exhaustive exercise program, the gastrocnemius and soleus muscles and blood samples from all rats were collected after anesthetization with Zoletil 50 (Virbac, France) and sacrifice. Each rat’s gastrocnemius and soleus muscles were removed and immediately frozen in liquid nitrogen for the measurement of glycogen. Muscle glycogen was determined using the method of Kuo et al. [15], in the following way: 50 mg of muscle was dissolved in 1 N KOH at 70°C for 30 min. Glacial acetic acid was added to dissolve the homogenate, and the mixture was incubated overnight in acetate buffer (0.3 M sodium acetate, pH 4.8) containing amyloglucosidase (Boehringer Mannheim, IN) and then neutralized by 1 N NaOH. Finally, samples were analyzed by measuring glucosyl units via the Trinder reaction (Sigma, MO, USA).

Blood samples (serum) were taken from the abdominal aorta, centrifuged at 1500 rpm for 15 min and analyzed for FFAs, blood glucose, and insulin levels. This was achieved using the following assay kits: BioVision (CA, USA) for FFAs, Sigma (MO, USA) for blood glucose, and Mercodia (Uppsala, Sweden) for insulin. All assays were performed in duplicate according to the procedures outlined in the manufacturers’ instructions and on the same day to reduce inter-assay variations. The intra- and inter-assay coefficients of variation (CVs) were 5% for FFAs, blood glucose, and insulin.

### Data analysis

All data were expressed as the mean ± standard deviation and were analyzed by SPSS software (SPSS vers. 15.0, Chicago, IL). A one-way ANOVA was performed for muscle glycogen, serum FFAs, glucose, and insulin. If the *F* value showed evidence of significance in the data, Tukey’s *post-hoc* test was used to identify where significance existed between groups. Because the exhaustive running was only performed in the ExSCP and Ex groups, this variable was analyzed by a Student’s unpaired *t*-test. The significant level was set at p > 0.05.

## Results

Before SCP supplementation, the body weights of the SD rats were similar in all three groups (203.4 ± 2.5 g for C, 204.1 ± 2.6 g for Ex, and 203.7 ± 2.6 g for ExSCP). Although the changes in the rats’ body weights occurred after the one-week experiment was completed, no significant differences were found across the three groups (217.0 ± 11.0 g for C, 221.9 ± 10.5 g for Ex, and 213.3 ± 10.9 g for ExSCP, measured before the exhaustive running).

**Figure 2:**
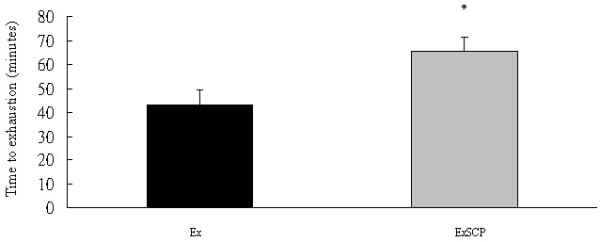
**Running time to exhaustion in the exercise (Ex) and exercise plus sweet cassava polysaccharide (ExSCP) groups.** *Significantly differs from the Ex group at p > 0.05.

The running times to exhaustion for the Ex and ExSCP groups were 43 and 64 min, respectively. This variable in the ExSCP group was significantly longer, by 49%, compared to the Ex group (Figure 2). This result matched the expectation of the priori statistical power calculation.

**Figure 3:**
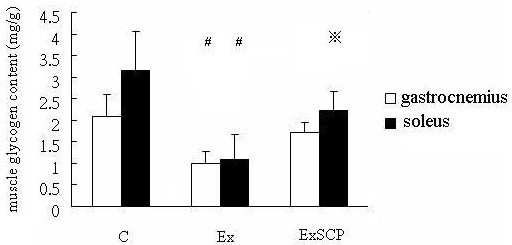
**Gastrocnemius and soleus muscle glycogen content in each group.** Notes: C, control group; Ex, exercise group; ExSCP, exercise plus sweet cassava polysaccharide group. #Significantly different from the C and ExSCP groups. ※Significantly different from the C group at p > 0.05.

The glycogen contents of the soleus muscle in the Ex group were significantly lower than in the ExSCP and C groups. In addition, those of the ExSCP group were significantly lower than the C group. The glycogen contents of the gastrocnemius muscle of the Ex group were significantly lower than those of the C and ExSCP groups, but no significant difference was evident between the C and ExSCP groups (Figure 3, Table 1).

**Table 1:** The muscle glycogen content of the gastrocneminus and soleus muscles in the three groups (post-exercise in the Ex and ExSCP group)

	**C**	**Ex**	**ExSCP**
Gastrocnemius (mg/g)	2.1 ± 0.5	1.0 ± 0.3^#^	1.7 ± 0.2
Soleus (mg/g)	3.1 ± 0.9	1.1 ± 0.6^#^	2.2 ± 0.4^※^

^#^Signifinantly different from the C and ExSCP groups.

^※^Significantly different from the C group at p > 0.05.

Notes: C, control group; Ex, exercise group; ExSCP, exercise plus sweet cassava polysaccharide group.

**Figure 4:**
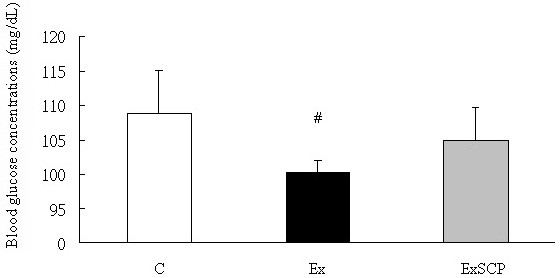
**Blood glucose concentrations in each group.** #Significantly different from the C and ExSCP groups at p > 0.05.

**Figure 5:**
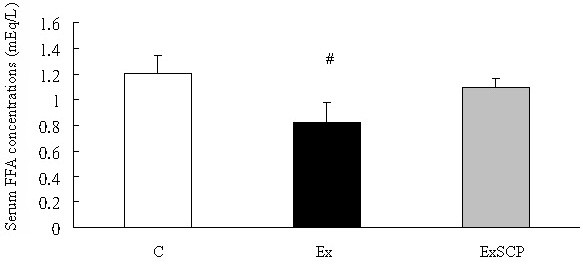
**Free fatty acid concentrations in each group.** #Significantly different from the C and ExSCP groups at p > 0.05.

**Figure 6:**
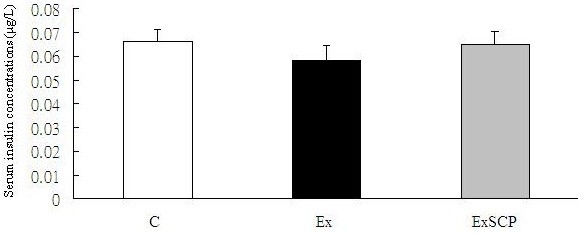
Insulin concentrations in each group.

Regarding the metabolites in the circulation, blood glucose levels in the Ex group were significantly lower than those in the ExSCP and C groups; no significant difference was found between the ExSCP and C groups (Figure 4). Similarly, the FFA concentration of the Ex group was significantly lower than that of the C and ExSCP groups, but no significant difference was evident between the C and ExSCP groups (Figure 5). In the case of insulin, no significant differences were found among the three groups, although the Ex group had lower concentrations compared to the other two groups (Figure 6, Table 2).

**Table 2:** The blood metabolites in the three groups (post-exercise in the Ex and ExSCP group)

	**C**	**Ex**	**ExSCP**
BG (mg/dL)	111.4 ± 5.6	100.1 ± 1.9^#^	109.1 ± 4.7
FFAs (mEq/L)	1.2 ± 0.1	0.8 ± 0.1^#^	1.2 ± 0.1
Insulin (μg/L)	0.064 ± 0.006	0.058 ± 0.006	0.064 ± 0.007

Notes: BG: blood glucose; FFAs: free fatty acids.

^#^Significant different form the C and ExSCP groups.

### Relationships

Data from all rats in the Ex and ExSCP groups were pooled together for the analyses of parameter correlations because significant correlationships were found between the soleus, gastrocnemius glycogen content and the running time to exhaustion in the respective exercise groups (data not shown). The running time to exhaustion correlated significantly with the soleus (*r*=0.65, p > 0.002) and gastrocnemius (*r* = 0.60, p > 0.004) glycogen content.

## Discussion

Despite cassava having a high carbohydrate content and potential benefit for sports performance, no study has investigated the effects of cassava on sports performance. As a result, this is the first study to examine the extracted polysaccharides from sweet cassava on sports performance in rats, to our knowledge.

The literature shows that muscle glycogen content is associated with running time to exhaustion in both human [16,17] and animal studies [18]. In addition, fatigue or a decline in sports performance is attributable to reduced muscle glycogen content [19,20]. As a result, increased muscle glycogen delays fatigue and/or extends the time to exhaustion. In this study, although muscle glycogen content in the soleus and gastrocnemius muscle was lower in the ExSCP and Ex groups compared to the C group after exhaustive exercise, the glycogen content in the two muscle types of the ExSCP group were significantly higher than that of the Ex group. This indicates that SCP supplementation may boost muscle glycogen. In addition, the ExSCP group had a longer running time to exhaustion compared to the Ex group, and the running time to exhaustion was also significantly related to muscle glycogen (*r* = 0.65 and 0.60 for the soleus and gastrocnemius muscles, respectively). Although these preliminary results were similar to those of the study by Bergstrom et al. [21], these findings should be interpreted cautiously, especially the causation between muscle glycogen and exhaustive performance, because this study did not measure the difference in muscle glycogen content between pre- and post-exhaustive exercise and we did not know how much muscle glycogen was metabolized during the exhaustive running. Further studies are necessary to address this issue.

Increasing muscle glycogen through diet, and before exercise, is one method of enhancing endurance capacity. Many researchers have tried to find new substances or regimes to elevate muscle glycogen in order to boost sports performance. Although some studies reported that sweet cassava has abundant carbohydrates, such as monosaccharides [2,3] and polysaccharides [4], little is known about whether there is any beneficial influence of sweet cassava on sports performance. In addition, several studies reported that supplementation with extracted polysaccharides is beneficial for increasing glycogen levels and extending the running time to exhaustion [10-12]. The effect of extracted polysaccharides from sweet cassava on boosting sports performance was similar to that seen in the above-mentioned studies and was proven in this study.

However, we cannot explain why the glycogen levels of the gastrocnemius muscles were different from those of the soleus muscles in the three groups. Because glycogen levels in the soleus and gastrocnemius muscles were not measured across the three groups before exercise, we do not know whether this difference already existed before exercise or was attributable to the physiological characteristics of the two muscles (i.e. slow-twitch fibers in the soleus muscle and fast-twitch (FT) fibers in the gastrocnemius muscle). This is one of the limitations of this study.

Blood glucose and insulin concentrations are important markers of carbohydrate metabolism during exercise. Regarding insulin, despite a tendency to be lower in the Ex group compared to the other two groups (p=0.054), this variable did not reach statistical significant. The maintenance of normal blood glucose levels during exercise by ingesting carbohydrate-containing foods before or during exercise can prolong the exercise time and delay fatigue [22-24]. In the present study, although the blood glucose concentrations were lower in the ExSCP group after the exhaustive exercise than in the C group, no significant difference was evident between these two groups. Additiionally, the blood glucose of the Ex group was significantly lower than that of the C and ExSCP groups. Several studies indicate that deteriorations in sports performance are related to hypoglycemia in several prolonged types of exercises [25-27]. As a result, maintaining euglycemia is crucial during the later stages of exercise. In this study, blood glucose concentrations after exercise in the ExSCP group were similar to those in the C group, but significantly higher than the Ex group. This result suggests that SCP supplementation benefited the maintenance of blood glucose levels.

Differences in FFA levels among the three groups were similar to blood glucose levels, with the FFA levels of the C and ExSCP groups being significantly higher than those of the Ex group; however, no significant difference existed between the first two groups. One study [28] has reported that elevated FFAs in the circulation can delay the onset of glycogen depletion and prolong exercise times. The current result is in line with this finding. However, other research [29,30] does not support the idea of increased FFAs being associated with the time to exhaustion or prolongation of endurance performance. Nevertheless, exercise intensity in the exhaustive exercise model was considered to mobilize more FFAs leading to higher muscle glycogen. The model of this exhaustive running was modified and inferred from the study of Brooks and White [13]. In the present study, the exercise intensity at 0% gradient with the same speed as the study by Brooks et al. might be lower than the estimated intensity (70%~75% VO_2_max). Lipids would be the main energy source during exercise of moderate intensity, especially FFAs in the circulation [31,32]. Lower exercise intensity in this study might account for the differences in muscle glycogen and FFAs. In addition, the assessment of the point of exhaustion might be another factor for this situation because we did not use other methods, such as electrical stimulation or external strong force. When rats displayed signs of exhaustion the exercise was terminated.

Finally, of all the measurable variables in this study, we only compared the Ex and ExSCP groups after exercise to the control group. No data for the muscle glycogen content or blood metabolites before exhaustive exercise were obtained in any respective group (especially the ExSCP and Ex groups) and this represents a major limitation.

## Conclusions

SCP, like other plant polysaccharides, can increase muscle glycogen content after supplementation. The maintenance of stable blood glucose and FFA levels with higher muscle glycogen, by means of SCP supplementation, contributed to extending the running time to exhaustion. Because liver glycogen is necessary for maintaining stable glucose levels in the circulation, further studies to examine the effects of SCP supplementation on liver glycogen are needed. Also, as mentioned the effects of differences between pre- and post-exercise muscle glycogen levels on running performance need clarification.

## Competing interests

The authors declare that they have no competing interests.

## Authors’ contributions

All authors contributed to the study design, the muscle and blood collection procedure, biochemical analyses, statistical analysis, and preparation of the manuscript. All authors have read and approved the final manuscript.
